# Who Is My Neighbor?

**Published:** 1888-10

**Authors:** 


### Who Is My Neighbor?

[From the American Lancet.]

Human beings touch at numerous points. Their brotherhood may be denied or ignored, but it perpetually reasserts itself. It is a truth of physical science as well as of Divine revelation that unto himself no man can live or die. In the matter of communicable diseases, this matter assumes an awful significance. Thus, recently a case was reported of a child, a member of a large family, who was taken sick with scarlet fever on reaching a large city far from home. In vain the parents sought in that city for a place, public or private, in which to care for this sick child. There was no such place, and the father was compelled to take his child in a crowded sleeping-car on a long journey. One shudders to think of the exposure to numerous persons, old and young, to the contagion of this disease. The child was a neighbor to these and doubtless to some it transferred the deadly poison.

Carlyle tells the story of a poor widow thus: Her husband died of typhus, in one of the lanes of Edinburgh, and she wandered about the town with her three children, seeking help, and finding none, returned to her lane and died there. Her journeying started an epidemic that killed seventeen persons. Those to whom this widow appealed denied her sisterhood to them, but she proved her sisterhood, as her typhus fever killed them.

Numerous instances are fresh in every mind, proving the same truth. Cholera, yellow fever, small pox, etc., are each constantly proving to the world that each individual may be a neighbor to one distant thousands of miles. It is dfficult to prove that any one individual in the world may not be a neighbor to any other person, by communicating to the same some diseased germ or poison. So completely has rapid transit annihilated time and space, that persons and articles carrying disease are met at most unlooked for places and under unwonted circumstances.

Admitting this, it is a question of personal import to every person that all other persons in the world be free from the contagium of disease. Hence, the one thing that should meet the heartiest support of all is the cleanliness of the person and abodes of the entire race. Unless this can be secured, commercial and social intercourse is fraught with danger.

What shall I do with my dirty neighbor? This is a question of the times, which all sanitarians are seeking to answer, and to which at another time we shall direct attention.

